# Computer-aided drug discovery

**DOI:** 10.12688/f1000research.6653.1

**Published:** 2015-08-26

**Authors:** Jürgen Bajorath

**Affiliations:** 1 Department of Life Science Informatics, Rheinische Friedrich-Wilhelms-Universität, Dahlmannstr. 2, Bonn, D-53113, Germany

**Keywords:** computational, drug discovery, in silico

## Abstract

Computational approaches are an integral part of interdisciplinary drug discovery research. Understanding the science behind computational tools, their opportunities, and limitations is essential to make a true impact on drug discovery at different levels. If applied in a scientifically meaningful way, computational methods improve the ability to identify and evaluate potential drug molecules, but there remain weaknesses in the methods that preclude naïve applications. Herein, current trends in computer-aided drug discovery are reviewed, and selected computational areas are discussed. Approaches are highlighted that aid in the identification and optimization of new drug candidates. Emphasis is put on the presentation and discussion of computational concepts and methods, rather than case studies or application examples. As such, this contribution aims to provide an overview of the current methodological spectrum of computational drug discovery for a broad audience.

## *In silico* drug discovery research

Various computational approaches are widely employed in the highly complex, time consuming, and resource-intense process of drug discovery ^1, 2^. Developing a new drug typically requires 10+ years and billion-dollar budgets. In the pharmaceutical industry, early- to mid-phase drug discovery efforts concentrate on advancing therapeutically relevant small molecules (or biologicals) and bringing candidate compounds into clinical trials. Computational methods are mostly, but not exclusively, applied during the early phase of drug discovery when basic research efforts aim at deciphering disease-related biology, prioritizing drug targets, and identifying and optimizing new chemical entities for therapeutic intervention. In general, primary goals of *in silico* approaches in drug discovery include the generation of better compounds with desirable *in vitro* and *in vivo* properties. Furthermore, computational analysis provides essential help in decision making and guidance for experimental programs, thereby reducing the number of candidate compounds to be evaluated experimentally. Since compound attrition rates in the clinic continue to be very high, on average ~90% for different therapeutic areas ^3^, a major challenge is trying to advance the best possible candidates to clinical trials. However, their ultimate success or failure continues to be unpredictable. Over the past three to four decades, the use of computational methods in drug discovery settings has steadily increased and computations have become an integral part of discovery research. Although drugs are not discovered and developed *in silico*—and predictions cannot alleviate the need for experimental work—computational approaches make valuable contributions to the highly complex discovery process at different levels. Hence, understanding opportunities and limitations of popular *in silico* approaches should be of considerable interest to a wide drug discovery and development audience.

In this contribution, recent advances in computer-aided drug discovery will be reviewed and put into perspective, highlighting unsolved problems and future growth areas. Rather than attempting to provide a comprehensive account of relevant *in silico* approaches, which would go much beyond the scope of this article, specific computational areas and current trends will be discussed.

## Classification scheme

**Figure 1.**
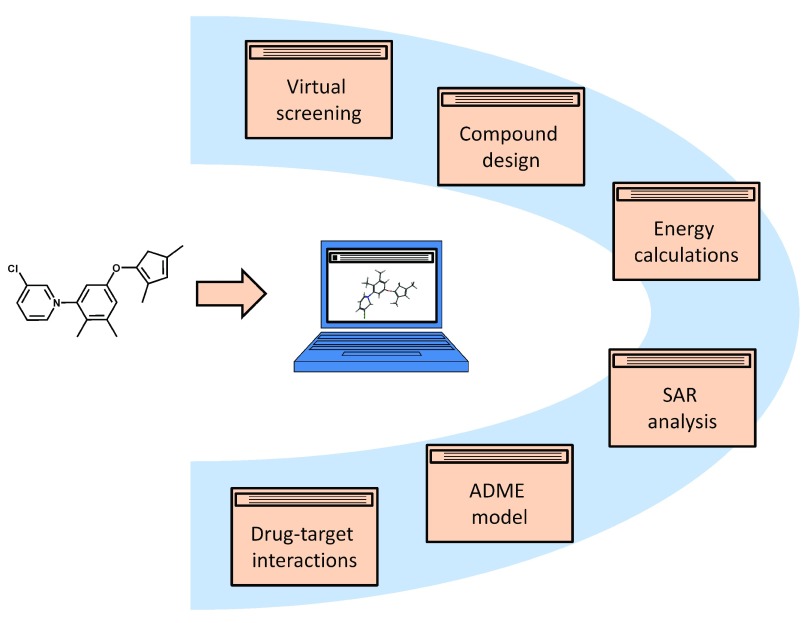
Areas of computer-aided drug discovery. Selected computational areas are shown providing focal points of the discussion. Each subject area covers a variety of computational approaches, as discussed in the text.

In general, *in silico* approaches with utility for drug discovery can roughly be divided into three major categories. These include the following: first, the design, implementation, and maintenance of computational infrastructures to process, organize, analyze, and store rapidly growing amounts of drug discovery data (e.g. compound library, biological screening, pharmacological, clinical, and literature data); second, methods to help identify, characterize, and prioritize biological targets and establish links between target engagement, biology, and disease (these approaches essentially fall into the domain of bioinformatics); and third, methods to help make better compounds and generate drug candidates. While all three categories are equally relevant for drug discovery and development, the following discussion will predominantly focus on the latter one, that is, the core of computer-aided drug discovery and design. Figure 1 summarizes computational areas that will be highlighted. The definition of subject areas is intentionally broad to provide a general overview. It should be noted that each area covers a variety of computational approaches. For example, “structure-activity relationship (SAR) analysis” includes numerical and graphical approaches as well as ligand- and target structure-based methodologies including, among others, the derivation of mathematical models of SARs or prediction and evaluation of compound binding modes. Similarly, “virtual screening” and “compound design” cover ligand- and structure-based approaches. “Energy calculations” include molecular mechanics, quantum mechanics, and combined approaches, for example, for conformational analysis, molecular geometry calculations, or affinity predictions. Furthermore, both “ADME (absorption, distribution, metabolism, excretion) modeling” and the systematic study of “drug-target interactions” involve the application of a variety of machine learning approaches and the derivation of predictive statistical models. A key point is that the current spectrum of computational concepts with relevance for drug discovery is extensive and complex. Providing a general overview inevitably calls for simplification.

There are other emerging computational areas that can only partly be covered herein due to size limitations including, for example, the derivation of knowledge from the rapidly growing amounts of increasingly complex and heterogeneous discovery data (which are also becoming available in the public domain) ^4, 5^. This challenges computational scientists in the pharmaceutical industry to integrate (proprietary) internal and available external data, but provides a significant opportunity to further increase the knowledge base for drug discovery research.

## Finding new active compounds

**Figure 2.**
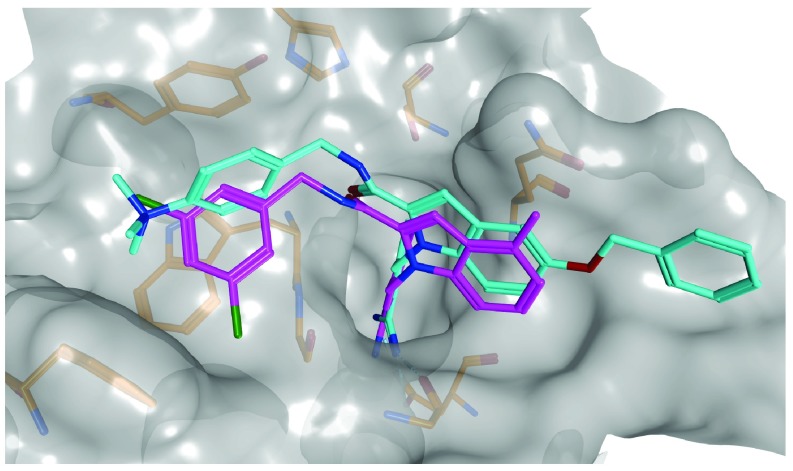
Binding mode prediction. In many instances, computer algorithms can predict correct or nearly correct interactions of small molecules and their target proteins. Shown are the X-ray structure of factor Xa (a serine protease) in complex with an inhibitor (cyan) and the putative binding mode of another inhibitor (magenta) generated by flexible ligand docking. The protein surface is rendered transparent (gray) and selected active site residues are depicted. The factor Xa inhibitors and X-ray structure were reported in 53.

For the identification of new hits, high-throughput screening is the primary approach in pharmaceutical research. For many years, biological screening has been augmented by computational compound database searching, so-called virtual screening ^6^, starting from known active compounds as templates (ligand-based virtual screening) and/or three-dimensional structures of target proteins (structure-based virtual screening). For ligand-based virtual screening, the molecular similarity relationship between known active and database compounds must be computationally explored ^7^; for structure-based virtual screening, test compounds are computationally screened on known ligand binding (active) sites of targets using docking calculations ^6, 8^. State-of-the-art ligand docking involves a conformational search of ligands within the structural constraints of active sites to model putative binding modes, followed by ranking of docked compounds according to their likelihood of activity. Ranking is based on computational scoring functions that approximate interaction energies. Figure 2 shows the X-ray structure of an exemplary enzyme-inhibitor complex and the putative binding mode of another inhibitor predicted by docking. Although virtual screening methods have a long history in computer-aided drug discovery, their accuracy is limited, mostly due to energy- or similarity-based scoring problems that have been known for many years, but are still not solved scientifically. In ligand-based virtual screening, calculated molecular similarity relationships (using different molecular representations and similarity functions) cannot be confidently correlated with observed activity relationships, representing a challenge for the identification of specifically active compounds. Furthermore, while the conformational search problem in docking is essentially solved, it is difficult to accurately rank compounds on the basis of force fields and energy functions and distinguish true positives (active compounds) from false positives. Despite these limitations, virtual screening studies have successfully identified many new hits for therapeutically relevant targets (including difficult screening targets) ^9– 11^. This is a situation often encountered in computational drug discovery. Computational methods typically have intrinsic limitations. Because it is hardly possible to fully and rigorously account for all physical and biological processes or phenomena on the computer, approximations need to be made at least to some extent. However, expert users typically know how to judge these approximations and evaluate the results of calculations taking their intrinsic limitations into account, which often leads to the successful identification of new active compounds. By contrast, naïve attempts fail more often than not, reflecting the fact that the application of *in silico* methods is far from being a routine process and requires a high level of expertise.

Given the uncertainties in identifying specifically active compounds in large databases (comparable to a “needle in a haystack” scenario) through molecular similarity analysis and/or docking, virtual screening cannot fully replace biological screening. Rather, virtual screening is expected to play out its full potential by closely interfacing with experimental screening and developing integrated (iterative) screening strategies ^12^. Iterative screening attempts to computationally prioritize small subsets of compound libraries for subsequent cycles of biological screening, taking information from newly identified hits into account. This iterative process reduces the number of library compounds for experimental evaluation and usually enriches active compounds in screening subsets. For these tasks, current virtual screening methods are sufficiently accurate. For example, state-of-the-art virtual screening makes it possible to prioritize 1–5% of the compounds from a large library having the highest probability of displaying a desired activity. Such calculations are also applied to generate target-focused compound libraries from large screening decks.

Although opportunities of combined computational and experimental screening were pointed out more than a decade ago ^12^, the implementation of iterative screening schemes has been slow in the pharmaceutical industry, in part owing to “philosophical” differences between experimental and computational screeners (i.e. whereas high-throughput screeners attempt to more efficiently process an increasing number of compounds, virtual screening proponents try to reduce the number of screening candidates as much as possible), and in part to technical difficulties (e.g. limited ability to cherry-pick compounds from screening plates). However, such technical limitations have largely been overcome, and recently there is increasing interest in integrated screening approaches ^13^. Hence, it will be interesting to observe future developments in this area.

In addition to virtual screening, new chemical entities are also generated by computational *de novo* design ^14, 15^, for which a variety of methods have been developed. Here, investigators often attempt to assemble compounds from fragments taking information from known actives into account or incrementally “grow” molecules within ligand binding sites. Similar to virtual screening, *de novo* design has its success stories, but also its pitfalls. A critically important criterion for the success of *de novo* design methods is the synthetic accessibility of proposed molecules. Therefore, a number of studies have attempted to incorporate reaction information (or retrosynthetic rules) into *de novo* design approaches.

## Lead optimization: evolving active compounds into drug candidates

Once new active compounds are identified, they enter the hit-to-lead and, subsequently, lead optimization phase, during which medicinal chemistry takes center stage. In the practice of medicinal chemistry, the cardinal question is “which compound(s) to make next” to further improve the potency and other drug-relevant properties (e.g. solubility, oral availability, or metabolic stability) of lead candidates. The generation of series of structurally analogous compounds with well-defined substitutions in the course of chemical optimization is often an essentially subjective process during which medicinal chemistry knowledge, experience, and intuition play a major role. Computational approaches in chemical optimization help to explore SARs and design analogs. Among these are, first and foremost, quantitative SAR (QSAR) methods that derive, at different levels of sophistication, linear SAR models from a series of active compounds to predict the activity of new analogs ^16^. QSAR analysis is an important component of computer-aided drug discovery, with scientific origins dating back to the 1960s, and a computational approach most medicinal chemists are familiar with. However, generating linear models of SARs on the basis of numerical property (descriptor) values is an approximation (as many SARs are non-linear in nature). Moreover, without a proper understanding of the statistical foundations of QSAR approaches, generated models are likely to be flawed. Taken together, both aspects help rationalize frequent pitfalls in prospective QSARs (i.e. incorrect activity predictions).

A major requirement for computational methods to efficiently support medicinal chemistry is that they are intuitive in nature and accessible to practicing chemists whose daily efforts are largely determined by the “which compound to make next” challenge. In recent years, several computational tools have been developed to specifically address this task. Among these are SAR visualization techniques ^17, 18^ that attempt to complement subjective analog design and numerical QSAR analysis with intuitive graphical representations to elucidate SAR patterns and identify key compounds for further exploration. This can be accomplished, for example, using molecular network representations in which compounds are represented as nodes and edges indicate similarity relationships ^18^. Such graphical representations make it possible to obtain global SAR views of compound data sets and also study local SARs formed by individual compound series.

Since lead optimization is a multi-objective process during which a variety of compound properties need to be balanced in addition to compound potency graphical SAR analysis becomes difficult in multi-property spaces. First attempts to visualize compound series in such multi-dimensional property spaces in a chemically intuitive manner are being made. In addition, multi-dimensional activity spaces are obtained when lead optimization sets are tested in different assays (against the same or alternative targets), which represents an equivalent analysis task. At the molecular level, visualization techniques can also be used to identify structural features in compound series that determine activity or other compound properties.

Another intuitive approach that is gaining increasing popularity in medicinal chemistry is provided by the concept of matched molecular pairs (MMPs) ^19– 21^. An MMP is defined as a pair of structurally similar or analogous compounds that are only distinguished by a chemical modification at a single site (i.e. the exchange of a substructure). MMPs are straightforward to understand from a medicinal chemistry perspective, can be systematically generated using efficient algorithms (even for large data sets), and make it possible to explore SARs and other compound-property relationships in a variety of ways ^19– 21^, always with a focus on chemical interpretability.

One of the major essentially unsolved problems in computer-aided drug discovery is the consistently accurate prediction of compound affinities. There is consensus in the field that the ability to reliably predict the free energy of binding for compounds in modeled or experimentally determined structures of ligand-target complexes would be a milestone event and put drug design up on a new level. This is an area where lead optimization, computational chemistry, interactive molecular modeling, and structure-based drug design meet, and the prediction of binding energies has been high on the scientific agenda for at least 20 years. To guide lead optimization and the design of new compounds, accurate prediction of absolute free energies and binding or dissociation constants would not be essential, as long as relative energies (differences in the free energy of binding) could be consistently obtained. Such predictions are mostly attempted by free energy perturbation calculations that mimic thermodynamic ligand inter-conversion cycles ^22, 23^. As is the case with docking, such perturbation calculations are also based on force fields that can only approximate ligand binding processes. Molecular mechanics force fields are often combined with explicit or implicit solvation models to further increase the physical basis of molecular dynamics simulations and their accuracy. Force field-based and quantum mechanical calculations are also combined to model protein-ligand interactions in greater detail.

Despite methodological limitations, success stories in predicting the relative free energies of binding have been reported for individual targets and analog series over the years ^22^, but the generality of such predictions has been questioned. For each apparent success, there have been failures (which are, however, rarely published). Although these calculations have been reported for decades, they have not been widely (or routinely) applied in the pharmaceutical industry due to their high computational expense and limited success. However, this is changing. In recent years, much emphasis has been put on further improving force fields and prediction methods ^23^. In addition, free energy calculations have also been supported by advances in molecular dynamics simulations of target proteins ^24^. Recently, increasingly consistent relative free energy predictions have been reported for different molecular systems ^25^. It will be interesting to monitor further progress in this area and see whether advanced methods are truly generalizable across therapeutic targets and compound classes and whether reliable predictions can be consistently achieved prospectively for compounds whose affinity is not yet known (different from typical validation calculations that are mostly retrospective in nature).

## Drug-likeness concept

Over the past two decades, another major topic of computational research and data analysis in drug discovery has been the concept of “drug-likeness” ^26, 27^, aiming to identify molecular features that are characteristic of drugs and set them apart from other bioactive compounds. This has indeed been one of the most intensely investigated concepts in computer-aided drug discovery, resulting in a plethora of publications reporting property distributions of drugs compared to other small molecules. Going beyond property-based rules, multi-objective optimization using machine learning techniques, desirability functions, and other quantitative measures of drug-likeness have also been introduced ^28– 31^. While property combinations have been identified that characterize classes of drugs or favor drug development we currently do not understand “what makes a drug a drug” or what distinguishes drugs from non-drugs. In fact, such questions might be difficult to address scientifically, taking into consideration that the selection of drug candidates is a multi-factorial process strongly influenced by criteria other than biological activity and efficacy including, among others, intellectual property, production, economic, or regulatory aspects. Nonetheless, exploring the concept of drug-likeness has yielded many guidelines concerning molecular properties that favor oral availability and *in vivo* efficacy of compounds without drawing a clear line between drugs and non-drugs.

## Balancing efficacy and safety

Closely related to the issue of drug-likeness is the prediction of ADME characteristics of compounds as well as toxic effects ^32, 33^. Given the high attrition rates during clinical trials, it is not surprising that the prediction of ADME properties is an intensely investigated topic in computer-aided drug discovery—and a truly challenging one. First and foremost, the molecular basis of the *in vivo* behavior of bioactive compounds and many toxic effects is currently only partly elucidated. In this context, the general rule applies that it is difficult, if not impossible, to derive reliable computational models for phenomena that we do not fully understand, such as the complex *in vivo* fate of drugs. Moreover, high-quality ADME data have typically been sparse, which has further complicated computational modeling ^34^. Better assay technologies with further improved reproducibility, more extensive compound profiling, and broader chemical space coverage are currently further improving the basis for ADME modeling in the pharmaceutical industry. Over the years, a variety of computational ADME investigations targeting specific molecular systems, such as, for example, drug-metabolizing enzymes, have been reported ^35– 37^, and modeling has produced some promising predictions of drug metabolism. Given its high relevance, there is little doubt that ADME analysis and the prediction of *in vivo* effects will continue to be a hot topic in computer-aided drug discovery, even if the scientific foundations are rather shaky at times. ADME modeling is an exemplary area where a close integration of experimental and predictive approaches is expected to yield further progress ^34^, similar to the situation with biological and computational screening, as discussed above. Large-scale prediction of molecular toxicity (beyond the detection of known toxic substructures) is likely to become an additional focal point of research in this field.

## Systematic assessment of ligand-target interactions

Another major growth area for computer-aided drug discovery is the systematic assessment of drug- and ligand-target interactions given the growing amounts of available data. Such analyses are often captured in drug-target networks ^38, 39^. In addition, predictive models of target activity can be derived on the basis of compound activity classes using machine learning methods ^40, 41^. These data mining and prediction exercises are highly relevant for the study of polypharmacology ^41– 44^ and the identification of secondary drug targets. Polypharmacology refers to increasing evidence that the efficacy of many drugs depends on interactions with multiple targets and simultaneous engagement of multiple signaling pathways, with protein kinase inhibitors applied in oncology being a prime example ^45^. Systematic accounts of ligand-target interactions have made it possible to predict unwanted side effects of drugs or candidates ^46, 47^ and identify previously unknown “positive” targets of existing drugs ^48– 50^. The latter aspect is of high relevance to finding new therapeutic applications for approved drugs, so-called drug repurposing or repositioning ^48^, which is currently another hot topic in the pharmaceutical industry. Ligand-based methods for target identification continue to be developed. For example, a computational approach has recently been introduced to predict the target of natural products on the basis of structural decomposition and fragment mapping to known drugs ^51^. Finally, pursuing an “inverse” polypharmacological route, it has also been possible to computationally design ligands with desired multi-target profiles on a large scale ^52^.

Approaches to systematically account for or predict ligand-target interactions are often of fairly low computational complexity, which provides a good example for the fact that the impact of computational approaches on drug discovery does not necessarily scale with methodological complexity or the magnitude of computations. Rather, asking questions that are particularly suitable for computational analysis or design is often the key.

## Looking ahead

As discussed herein, computational approaches in drug discovery are mostly used to tackle rather challenging tasks. Thus, it should not come as a surprise that success is limited at times. In addition, a number of fundamental computational problems that have been on the agenda for decades still remain to be solved. In the practice of drug discovery, it is of critical importance not to overestimate the potential of computational methods. This is harmful because it affects the credibility of serious computational work, which is without any doubt carried out in both academia and the pharmaceutical industry. In many instances, computational approaches can significantly advance discovery projects if they are carefully chosen and applied to problems that can actually be solved, such as the selection of database compounds with above-average probability to display a specific activity, identification of key compounds for chemical optimization, or design of ligands that favorably interact with a given binding site, to name just a few. The further development and impact of certain computational approaches will also likely differ in academic and industrial environments. For example, virtual compound screening approaches will be more important in academic settings where biological screening capacity is often limited. By contrast, the development of new computational methods for the analysis of data from phenotypic screening assays will be of prime relevance in the pharmaceutical industry. Regardless, it is hoped—and not unlikely—that further progress will be made in the coming years in key areas of computer-aided drug discovery including, for example the ranking of database compounds according to probabilities of activity or the prediction of compound potencies affinities. Moreover, there are other important issues to address. For example, the variability of biological assays and activity measurements (data heterogeneity and reliability) must be carefully considered in compound optimization for which systematic computational analysis of experimental data and data reliance assessments are pre-requisites. For complex biological screening systems, the identification of targets of active compounds (target de-convolution) will strongly depend on computational approaches. While first efforts in these directions have already been made, these types of applications are growth areas for computational drug discovery. Furthermore, reducing human bias in the generation and evaluation of molecular property spaces for ADME analysis and lead optimization will require further computational research. Last but not least, as the big data era enters drug discovery research, the development of novel computational concepts for analysis, organization, integration, and utilization of biological and chemical data will be essential. Going forward, cloud computing is expected to play a major role in handling big data. These are challenging and exciting times for computer-aided drug discovery. It is the view of the author that progress in computational drug discovery will continue to be evolutionary, rather than revolutionary (given the history of the field over the past two to three decades), but this does in no way reflect a pessimistic view. Incremental advances might have a substantial impact on the practice of drug discovery (e.g. considering the development of energy functions or conformational sampling techniques). For the future of computer-aided drug discovery, it will also be important to put further emphasis on the development of computational methods that are chemically intuitive and accessible to a wide discovery audience, beyond computational experts. In general, such methods have the greatest potential to be widely applied in the practice of drug discovery, a pre-requisite for success. If we consider that computer-aided drug discovery continues to be driven by experts, as discussed above, a major step forward for this field would indeed be, the generation of chemically intuitive and robust computational methods that become an integral part of day-to-day discovery efforts.

## Abbreviations

ADME, absorption, distribution, metabolism, excretion; MMP, matched molecular pair; SAR, structure-activity relationship; QSAR, quantitative structure-activity relationship.
